# Supplementary material for: Evaluating the method reproducibility of deep learning models in biodiversity research
Source: PeerJ Comput Sci. 2025 Feb 5;11:e2618. doi: 10.7717/peerj-cs.2618 (PMC11888858; doi:10.7717/peerj-cs.2618)
Supplement: Supplemental Information 2 [file peerj-cs-11-2618-s002.pdf]

https://doi.org/10.5281/zenodo.13987177
